# On the Treatment of Malarious Fever by the Subcutaneous Injection of Quinine

**Published:** 1863-11

**Authors:** W. J. Moore

**Affiliations:** Bombay Medical Service; Bombay


### ON THE TREATMENT OF MALARIOUS FEVER BY THE SUBCUTANEOUS INJECTION OF QUININE.

By W. J. MOORE, L.R.C.P.Ed., Bombay Medical Service.

Reprinted from the "London Lancet."

Since the year 1858, when Dr. Wood brought forward the hypodermic method of administering morphia, the plan has been extensively tried. Moreover, the results following the injection of morphia into the subcutaneous areolar tissue have, on the whole, been satisfactory, and the use of the alkaloid in this manner has now become an established practice in various obstinate neuralgic disorders. Other agents, as atropia, have also been used hypodermically with varied success, and I have latterly employed a strong solution of quinine for the cure of intermittent and remittent fever by the method of subcutaneous injection.

The success which has attended the practice renders me desirous of calling attention to this novel mode of using quinine. I have so employed the remedy in upwards of thirty cases of intermittent fever, and in several cases of remittent, and with almost invariable success, the former class seldom requiring a second application, the latter generally subsiding after the fifth or sixth injection. Since the period I commenced to use quinine in this manner, I have been surprised and pleased to find in one of the medical periodicals that the same plan has been pursued by Dr. Chasseaud, of Smyrna, who reports 150 cures, and especially recommends the system in fever complicated with gastric symptoms, when the exhibition of quinine by the mouth is often "inefficient, difficult, and hazardous."

I use the strongest solution of quinine which can be prepared —viz., thirty grains of quinine, eight or ten drops of dilute sulphuric acid, and half an ounce of water. Of this I inject from half a drachm to a drachm, the former quantity containing some four grains of the active agent. With the exception of a little sulphate of soda, if the bowels are confined, I use no other remedies whatever in uncomplicated cases of any type of malarious fever. When the spleen is enlarged, or if a leucocythemic condition is present, I prescribe, as an additional curative agent, one or other of the preparations of iron—very frequently the citrate of iron and quinine.

I generally inject beneath the skin over the outer belly of the triceps extensor muscle, and sometimes over the deltoid. I have, however, used the syringe with equal effect on the thigh and calf, and in cases of enlarged spleen have thought the action of the remedy increased by injecting over that organ. I use a small glass syringe with the screw action, and furnished with a sharp silver point some half an inch in length. The latter is introduced beneath the integument half an inch or less, and the pain is not greater than the prick of a pin. Indeed, patients have frequently declared they would rather submit to this process than taste the bitter of quinine. I have never seen the slightest inflammation or irritation follow the operation except in two instances. In one of these this result was due to the instruments employed—namely, a small trocar and common glass syringe; in the other, to quinine in *suspension* being used instead of in *solution*. Indeed, I have reason to think that quinine in suspension is very irritating to the tissues, and this is what physiology would lead us to expect, as it is certain that when a fluid material is introduced into the areolar structure, it will be absorbed more directly than any solid mass could be. Therefore, to avoid irritation of the parts, and, also, to prevent "choking" of the syringe (and which instrument was procured from England), I insist upon a perfectly clear solution of the alkaloid.

The best time to inject is shortly before the expected cold fit, but it may be done during the first stage with the effect of lessening and somewhat stopping the whole paroxysm. Latterly when a patient presents at the morning visit, who expects an accession during the day, I have injected at the time, and nearly invariably the fever has stopped.

In cases of remittent I have endeavored to inject during the remission, but do not wait for this period. In severe cases the injection should be repeated at intervals of six or eight hours.

I believe four or five grains of quinine injected beneath the integument are equal in their effects to five or six times that amount taken into the stomach; also, that the effects are more certain than when taken in the ordinary method; and, also, that relapsing attacks are less common than when the remedy is administered by the mouth.

Bombay, 1863.

